# Disability, gender, and employment relationships in Africa: The case of Ghana

**DOI:** 10.4102/ajod.v4i1.95

**Published:** 2015-06-03

**Authors:** Augustina Naami

**Affiliations:** 1 Department of Social Work, College of Behavioral Sciences, University of Northern Iowa, United States of America

## Abstract

The exploratory quantitative study sought to develop an understanding about the relationships among disability, gender and employment in Northern Ghana. A total of 110 individuals with disabilities (20–60 years) from various disability groups participated in the study. The results indicate that many persons with disabilities are unemployed, the majority being women. Discrimination is cited as the greatest barrier to the employment of persons with disabilities, particularly women. The majority of persons with disabilities, typically women, live in poverty; given that some are unemployed and those who are employed worked mostly in marginal, seasonal and menial jobs. Persons with disabilities also experience several challenges on the job, including negative perceptions about their capabilities, discrimination and exclusion, irrespective of the employment sector and disability type. Educational interventions such as workshops, documenting and showcasing success stories of persons with disabilities could be helpful to reduce negative perceptions about their capabilities as well as discrimination against them. Government intervention to support persons with disabilities with start-up capital and funding for formal education is also recommended as these two elements were identified respectively as barriers to self-employment and employment in the public/private sectors. Government interventions to create educational opportunities for persons with disabilities are essential given that lower educational attainment affect their employment.

## Introduction and literature review

Employment related disparities persist in both develop and developing countries. Women, in general, are less valued in the labour market, as shown by inequality in participation in the labour force, pay, the kind of jobs they have, and the positions they hold. Several global employment studies (Azmat, Guell & Manning 2006; Hausmann, Tyson & Zahidi 2013; United Nations 2013a, 2013b, 2013c; United Nations Economic Commission for Europe [UNECE] 2008) have converging findings regarding gender employment related disparities.

In 2012, the United Nations reported that the percentage of adult women in the labour force is lower than their male counterparts in all the United Nations’ countries. The report emphasises consistency in the global gender employment gap over several different years. For example, between 2002 and 2007, female unempolyment rates remained 5.8%, 0.5% points higher than their male counterparts (5.3%). It is worth mentioning that women from Africa, Asia and Latin America experienced higher uemploymment rates (United Nations 2013b). The 2012 report further notes the widening global gender employment gap.

Studies also emphasise the persistency of the gender pay gap. Available statistics indicate that the current global average gender pay gap (which is the difference in earning between men and women) is hovering around 17% (United Nations 2013c). There is also evidence that women are underrepresented in official and managerial positions, whilst they are overrepresented as sales, clerical, and service workers. The UNECE in 2008 found that Europe and North America has a clear majority of men among legislators, managers and senior officials, except for the United States and Lithuania, where the proportion of women among legislators, managers, and senior officials was rather high (54% and 47% respectively).

Also, various United Nations reports highlight the overrepresentation of women in the service sectors in both developed and developing countries (United Nations 2013a, 2013b, 2013c). Many other women are in vulnerable employment; working for family or self (United Nations 2013a). Vulnerable employment is characterised by working mostly in temporal, low paying marginal or underemployment, seasonal and menial jobs. They also lack job benefits such as social security and health insurance. The average global vulnerable employment gender gap is estimated at 2.3%, with a larger share of women in vulnerable employment (50.4%) compared to men (48.1%). The vulnerable employment gap reported for Sub-Saharan Africa (of which Ghana is a part) was rather high, estimated at 15%.

Similar gender employment disparities persist in Ghana. Although available statistics (Ghana Statistical Service (2006) show that males’ employment rate (54.9%) is just slightly higher than that of females (53.4%), women are more likely to engage in vulnerable employment (females [71.3%], males [37.9%]), and less likely to work in paid employment, given that the proportion of males in paid employment is much higher (25.0%), compared to that of females (8.2%). Furthermore, there is inequality in the pay men and women receive. Men receive higher earnings (61 Ghana Pesewas [Gp]) than females (50 Gp). The average hourly earnings are 55Gp (1.96 Ghana Cedis [GhC] = US$1 in 2012). Educational levels are also higher among men (67%) than women (46%) (Food and Agricultural Organisation [FAO] 2012).

Parallel to the precceding discussions, global employment disparities exist between persons with and those without disabilities. In their study, which gives a snapshot of economic and poverty situation of persons with disabilities of working age in 15 developing countries, Mitra, Posarac & Vick (2011) charted several pathways relating disability to poverty. They conclude that persons with disabilities, compared with persons without disabilities, are more likely to have lower educational attainment, experience lower employment rates, have lower wages when employed, and are more likely to be poor. They however, note that the intensity of the association of disability and poverty depends on contextual factors including individual, family, community, and country.

A global disability employment gap is evident in several other studies. Employment rates for individuals with disabilities are lower than those without disabilities (Dhungana 2006; Groce *et al.* 2013; Heymann, Stein & Moreno 2013; Mitra 2006; Mitra & Sambamoorthi 2008, 2009; Mizunoya & Mitra 2012; Ozawa & Yeo 2006; World Health Organization Report on Disability 2011). The result of The World Health Organization Survey on Disability Report for 51 countries shows employment rates of 52.8% for men with disabilities and 19.6% for women with disabilities, while that for men and women without disabilities were respectively 64.9% and 29.9%. Also, Mizunoya and Mitra, whose study covered 15 developing countries, including Ghana, investigated disability gaps in employment rates. They found that persons with disabilities have lower employment rates than persons without disabilities in 12 out of the 15 countries. They also found that persons with disabilities are more likely to work in the informal sector compared to their counterparts without disabilities. Their study showed a significant difference between workers with disabilities who are self-employed and their counterparts without disabilities in nine out of the 15 countries.

Other researches indicate that persons without disabilities earn significantly more than those with disabilities (Heymann *et al.* 2013; Kaye 2009; Mitra & Sambamoorthi 2008, 2009; Ozawa & Yeo 2006; World Health Organization Report on Disability 2011). For example, a study of 27 industrialized countries by McMahon *et al.* (2005) showed an average earning gap of 15%. Again, persons with disabilities who are employed mostly work in entry-level positions that do not utilise much of their skills. They also have difficulties climbing the corporate ladder to work in managerial and official positions (Barisin, Benjak & Vuletic 2011; Kaye 2009).

Disability and gender interact to create multiple disadvantages for women with disabilities compared with men with disabilities as a result of sexism and disabilism, discrimination against women and persons with disabilities respectively. Various studies (with different mesasures in both high, middle and low income countries) have converging results indicating higher disability prevelence among women with disabilities of working age compared to their male counterparts (Mitra & Sambamoorthi 2014; Mitra *et al.* 2011; Mizunoya & Mitra 2012; World Health Organization Report on Disability 2011). Mitra & Sambamoorth whose research sought to estimate disability prevalence among adults in 54 countries globally, found that women in all age groups in all the countries studied have higher disability prevalence than men. Mistra *et al*. (2011) found similar results from their study of 15 developing countries.

And, as in the scenario of the general gender employment disparity discussed previously, women with disabilities are not only less likely to be employed (Dhungana 2006; Randolph & Anderson 2004; Smith 2007), they also are more likely to receive lower pay (Ozawa & Yeo 2006) and are underrepresented in official and managerial positions, while they are overrepresented as sales, clerical, and service workers (Jans & Stoddard 1999; Smith 2007).

In developing countries the employment situation could be worse for both men and women with disabilities due to lower educational levels (Mitra et. al. 2011; Mizunoya & Mitra 2012; World Health Organization Report on Disability 2011), cultural beliefs and practices, negative perceptions about their capabilities (Naami 2014; Naami & Liese 2012), physical barriers and inaccessible transportation (Heymann *et al.* 2013; Naami 2014; Tijm, Cornielje & Edusei 2011). For women with disabilities, it could even be more complicated as a result of the intersection of disability and gender. However, there is dearth of literature to estimate the scope and trend of the employment situation of persons with disabilities, especially in Ghana where there is virtually no data about disability in national surveys.

The few studies that exist demonstrate a disability employment gap in developing countries (Mitra *et al.* 2011; Mizunoya & Mitra 2012). SINTEF studies of living conditions among people with disabilities in seven Southern African coutries, led by Arne Henning Eide, indicate the prevelence of disability and gender employment disaprities in all these countries except Zimbabwe (Eide 2013). This difference, however, reflects only formal employment. But, as discussed previously, persons with disabilities are more likely to work in the informal sector. Studies by both Mitra *et al.* (2011) and Mizunoya & Mitra 2012 indicate a disability employment gap in the case of Ghana. Mizunoya & Mitra further demonstrate a gender employment gap, though not statistically significant. However, there is dearth of literature to estimate the scope and trend of the employment situation of persons with with disabilities in Ghana. This research fills that gap.

It is noteworthy that Ghana made a landmark commitment to promote the rights of persons with disabilities when it ratified the Convention on the Rights of Persons with Disabilities (CRPD) in August of 2012. The CRPD is an international convention with 50 articles addressing various aspects of disability rights including non-discrimination, equality of opportunity, accessibility, respect for inherent dignity, full and effective participation, and inclusion as well as rights for women and children with disabilities. The treaty seeks to:

… promote, protect and ensure the full and equal enjoyment of all human rights and fundamental freedoms by all persons with disabilities , and to promote respect for their inherent dignity. (United Nations 2006:4)

Ghana has yet to adapt its policies to reflect the treaty in order to benefit persons with disabilities.

### 

#### Study purpose and objectives

The exploratory quantitative study sought to develop an understanding of the employment situation of persons with disabilities in Northern Ghana and to make recommendations for the government and other stakeholders in disabilities about ways to advance their employment. Specifically, the study sought to determine:

1. Which persons with disabilities are employed, what they do, the income they earn, and the nature of their work environment.
2. Which persons with disabilities are unemployed, barriers to their unemployment and how they provide for themselves and their families, given that social protection programs are virtually non-existent in Ghana.
3. Gender differences in the variables identified, and
4. Recommendations to improve on the employment of persons with disabilities.

## Methods and materials

The study used an exploratory descriptive quantitative design. The study design is imperative, since not much research is done in this area. The emphasis of the exploratory aspect of this design is to gain new insights, ideas and increase knowledge of all aspects of the social phenomenon (Brink & Wood 1998). The descriptive feature describes the frequencies at which the various variables occurred and how they vary to help our understanding of the phenomenon. One hundred and ten (110) persons with disabilities of working age from the three regional capitals of the Northern Sector of Ghana; Tamale, Wa, and Bolga participated in the study.

### Procedure

Participants were recruited from the Resources Centers for Persons with Disabilities, establishments that oversee the various organizations of persons with disabilities in the regions: namely Ghana National Association of the Blind, Ghana National Association of the Deaf and the Ghana Society of the Physically Disabled. Leaders of the disability movements generated lists of eligible members from existing records. From their lists, members were contacted by phone to volunteer to participate in the study. The volunteers were given adequate information about the study and were told that participation was completely voluntary. All study participants signed consent forms before the face-to-face interviews. The consent form was translated in the local language to enable those participants who felt more comfortable to express themselves in the local language to have the opportunity to do so. Both sign and local language interpretations were also provided for individuals with hearing disabilities and for those participants who would rather do the interview in the local language. The interviews lasted approximately 30 minutes each. Participants were given approximately seven GhC each (approximately $US5 at the time of the study) for participating in the study. Two research assistants were trained to assist in the data collection. The Institutional Review Board of the author’s institution approved this study.

### Measurement

The questionnaire was particularly designed for this study and had 45 questions in all. Literature about the various employment issues was searched for concepts that needed to be measured, which were included in the questionnaire. While some questions were closed-ended and a few Likert-type questions, the majority of the questions were open-ended questions soliciting detailed information about certain variables which were later recoded for descriptive and other statistical analysis. The questionnaire had three major sections: demographic information, employment, and income or support. The demographic information section comprised of variables such as age, gender, marital status, disability type, and educational levels, and contained nine questions in all. The section on employment addressed issues about both employment and unemployment of persons with disabilities. Issues such as the types of jobs persons with disabilities do, the nature of their work environment, challenges they encounter at work, and their recommendations to improve on their employment were also addressed.

Those who were unemployed were asked about barriers to their employment and their recommendations for support to find jobs. There were 26 questions in this section. The final section solicited information about participants’ income and the kinds and quality of support they receive as well as their present needs, 10 questions in all.

### Data analysis

Statistical Package for Social Sciences (SPSS) version 15.0 was used for the data analysis. Descriptive statistics and several other statistical tests (Mann-Whitey U, Kruskal-Wallis, and Chi-square tests) were used in the data analysis. The Chi-square test for independence was vital to analyse the categorical variables (e.g. length of unemployment/sex, education levels/sex, education/employment, sex/barriers to employment, sex/income). To compare the difference between three groups (e.g. disability groups, study locations and employment sectors), the Kruskall-Wallis test was the appropriate statistical test. And the Mann-Whitney U test compared the difference between men and women on variables such as income. Since the majority of the questions were open-ended, they were first recoded for descriptive and other statistical analysis. For example, categories of length of unemployment, income, support, barriers to employment, problems experience on the job were all developed from the open-ended questions. The researcher and research assistants separately recoded the variables, then compared and reviewed results to arrive at variables included in the analysis. Non-parametric statistics were used in the data analysis, since the sample has non-probability.

## Results

The four emerging themes based on the literature and data are: (1) unemployment, (2) employment, (3) income, and (4) support. Gender and disability differences are also reported under each theme as much as possible.

### Unemployment

This section describes persons with disabilities who were unemployed, barriers to their employment and their recommendations to find jobs. Unemployment in this paper refers to those who have no jobs but have been looking for jobs in the government, private and self-employment sectors irrespective of the length of job search. Although self-employment is part of the private sector, it is differentiated in this study for clearer discussion. The private sector refers to persons with disabilities working for other people and organizations other than for themselves.

The study outcome shows that about a quarter 27 (24.8%) of the respondents were unemployed. Unemployment rates were higher for women with disabilities 16 (59.3%) than for men with disabilities 11 (40.7%), but relatively the same for the three interview location: Bolga (9; 34.6%), Tamale (9; 34.6%) and Wa (8; 30.8%). However, more individuals with visual disabilities (11; 40.3%) were unemployed compared with those with hearing disabilities (11; 33.3%) and those with physical disabilities (7; 26%). The length of unemployment ranged between 1 and 20 years, with an average of 2.21 years, SD = 1.179. As seen in Table 1, almost half (10; 41.7%) of those without jobs have been unemployed between 6 and 10 years plus. An additional 5 (20.8%) have been unemployed for over 20 years. A Chi-square test for independence was conducted to assess the statistical difference between men and women on the categories of lengths of unemployment (1–5 years; 6–10 years; 10 years and above; and 20 years and above). The result of the test was significant, *x*^2^ (3, *N* = 24) = 9.5, *p* = .023, which means there was a difference in proportion between men and women on categories of length of unemployment. A follow-up test revealed that the proportion of women with disabilities on the 1–5 years category of length of unemployment significantly differ from the proportion of men, while that of men on the category of 10 years plus differ significantly from the women. However, there was no difference on category of length of unemployment for the various disability types.

**TABLE 1:** Length of unemployment by sex (*N* = 24 Females 13 and males 11).

Length of unemployment	Male (%)	Female (%)	Total (%)
1–5 years	9.0	61.5	37.5
6–10 years	27.3	23.1	25
10 years plus	36.4	0	16.7
20 years plus	27.3	15.4	20.8
Total	100	100	100

Respondents who were unemployed were asked to indicate if they had difficulties finding jobs. All of them answered in the affirmative. They mostly identified discrimination, inadequate start-up capital and skills as barriers to their employment. However, discrimination, which is defined in this study as the unfair treatment of those with disabilities as a result of their impairment, was cited as the key barrier to their employment. As seen in the cross tabulation in Table 2, although a few more females experienced discrimination and the other barriers than their male counterparts, the result was not statistically significant. Skills in this context refer to vocational or technical training.

**TABLE 2:** Barriers to employment by sex (*N* = 26 Females 15 and males 11).

Barriers	Sex		
	Male (%)	Female (%)	Total (%)
Formal sector	28.7	31.1	30.8
Self-employment	18	22.2	19.2
Lack of start-up capital	53.3	44.4	46.2
Inadequate skills	0	2.7	3.8
Total	100	100	100

It is noteworthy that formal education did not significantly (*x*^2^ (6, *N* = 110) = 6.89, *p* = .33) affect the employment of persons with disabilities in this study, given that the majority had technical/vocational education as indicated in Table 3. However, educational levels were significantly *x*^2^ (6, *N* = 110) = 12.6, *p* <.05 higher among male than female participants. To address their employment needs, the participants recommended further education and awareness creation about their capabilities for employment in the government sector, and start-up capital, stores and public education against discrimination for self-employment.

**TABLE 3:** Level of education by sex (*N* = 110 Females 60 and males 50).

Level of education	Sex		
	Male (%)	Female (%)	Total (%)
Elementary and junior high	14	21.7	18.2
High school	22	15	18.2
Technical/vocational	36	38.3	37.3
Teacher training college	2	3.3	2.7
Undergraduate	14	1.7	7.3
Graduate	4	0	1.8
No formal education	8	20	14.5
Total	100	100	100

### Employment

The employment section describes persons with disabilities who were employed, where they worked, the kinds of jobs they did, and the nature of their work environment.

Three out of 4 of the respondents (83; 75.2%) said they were employed. However, more than half (52; 64.2%) of them were self-employed; working mostly in marginal, seasonal and menial jobs. Surprisingly, more females (44; 53.7%) than males (39; 46.3%) pointed out that they were employed. But the women (32; 72.7%) dominate the self-employment sector compared to the men (20; 51.1%). The results also indicate that many individuals with physical disabilities (30; 57.7%), compared with people with visual disabilities (13; 25%) and those with hearing difficulties (9; 17.3%) were self-employed.

Furthermore, about one-quarter (21; 25.9%) of those who indicated they work worked for the government. The majority of this population was teachers (15; 71%). Many men with disabilities (9; 35.1%) worked in this sector than women (6; 18.2%). Also, more people with visual disabilities (12; 57.1%), compared to those with physical disabilities (6; 28.6%) and people with hearing disabilities (3; 14.3%) were hired by the government. The private sector, which employed (8; 9.9%) of the respondents, hired more people with hearing disabilities (5; 71.4%) than those with physical disabilities (2; 28.6%), but not individuals with visual disabilities. (Table 4)

**TABLE 4:** Employment sector by sex (*N* = 81 Females 44 and males 37).

Employment sector	Sex		
	Male (%)	Female (%)	Total (%)
Government	35.1	18.2	25.9
Private	10.8	9.1	9.9
Self-employed	54.1	72.7	64.2
Total	100	100	100

Regarding problems experienced at work, respondents employed in all three sectors (private, government and self-employment) identified negative perceptions about their capabilities and exclusion. The self-employed further identified inadequate funding for their businesses, delayed and/or no payment for services, lack of stores for their businesses, and marketing difficulties, while those in the government and private sectors pointed out the lack of accommodation on the job. To address these problems, respondents recommended further education, accommodation at work (such as assistive technology), accessible environment, funding for small businesses, marking their products and public education about their capabilities.

Participants who were employed were asked if they had ever received training on the job. Over half (15; 65.2%) of those working in the government sector reported having received in-service training for skills upgrade. But twice as many males with disabilities as females received training, as indicated in Table 5. On the other hand, approximately one- third (8; 34.8%) said they had never received training. Again, they were asked to indicate if they had ever been promoted. More than half (10; 55.6%) answered in the negative. Some individuals, however, answered in the affirmative (8; 44.4%). The majority (5; 41.7%) of these individuals is men with disabilities compared to women (3; 37.5%).

**TABLE 5:** Training and promotion of workers in the government sector by sex (%).

Training and promotion	Sex			
	Male (%)	Female (%)	Total (%)	*N*
Training	66.7	62.5	65.2	15 (M=10, F=5)
Promotion	41.7	37.5	44.4	8 (M=5, F=3)
N	15	8		23

Regarding job satisfaction, a Kruskal-Wallis test indicates that the self-employed were happier and more satisfied with their jobs than those working in the government and public sectors. The test was significant, *x*^2^ (2, *N* = 81) = 13.1, *p* = .001. Almost all the self-employed 94.9% (45; 5 missing cases) strongly agreed or agreed to the statement ‘I am proud and happy to be self-employed’, whilst only 23 (72.4%) of those working in private and government sectors strongly agreed or agreed to a similar statement ‘I am proud and happy to work for this organization’. However, just two (4.3%) of the self-employed disagreed to the same statement ‘I am proud and happy to be self-employed’ whilst 8 (27.6%) of those working in the private and government sectors strongly disagreed or disagreed to a similar statement ‘I am proud and happy to work for this organization’.

### Income

What income do persons with disabilities who work receive and from what sources? How do the unemployed provide for themselves and their families, given that social protection programs are virtually non-existent in Ghana? This section addresses these issues. See Tables 6–8 for the results of participants’ income.

**TABLE 6:** Respondents monthly income by sex (*N* = 105 Females 58 and Males 47).

Income in Ghana Cedis	Sex			
	Male (%)	Female (%)	Total (%)	*N*
2–80 GhC	70.2	87.9	80	84 (M=33, F=51)
81–200 GhC	12.8	6.9	9.5	10 (M=6, F=4)
201–300 GhC	2.1	0	1	1 (M=1, F= 0)
301–400 GhC	2.1	3.4	2.9	3 (M=1, F=2)
400–1100 GhC	12.8	1.7	6.7	7 (M=6, F=1)
Total	100	100	100	

1 96GhC=US$1 at the time of the study.

**TABLE 7:** Respondents monthly income by Sector (%) (*N* = 81 G 21, P 8 and S 52).

Income in Ghana Cedis	Employment sector			
	Government (G) (%)	Private (P) (%)	Self-Employment (S) (%)	Total
2–80 GhC	8.1	8.1	83.9	100
81–200 GhC	75	25	0	100
201–300 GhC	100	0	0	100
301–400 GhC	100	0	0	100
400–1100 GhC	85.7	14.3	0	100

**TABLE 8:** Respondents sources of income by sex (*N* = 105 Females 58 and Males 47).

Income source	Sex			
	Male (%)	Female (%)	Total (%)	*N*
Family/Friends	16.3	12.5	14.3	15 (M=8, F=7)
Self-employment	46.9	64.3	56.2	59 (M=23, F=36)
Government	24.5	16.1	20	21 (M=12, F=9)
Private sector	2	0	1	1 (M=1, F=0)
Begging	10.3	7.1	8.5	9 (M=5, F=4)
Total	100	100	100	-

Participants’ monthly income ranged between 2–1100 Ghana Cedis (approximately US$1–561USD), mean income is GhC 1.47 per day (which is less than US$1, SD = 1.119). The majority (84; 80%) of the respondents earned just a little over US$1 (US$1.36, the minimum daily income reported in this study). An additional 10 (9.5%) earned US$3.4 daily. It is therefore not surprising that almost all the participants 106 (96.4%) emphasised that their income was not enough to provide for their basic necessities, with only 5 (3.6%) responding to the contrary.

As indicated in Table 6, almost all the female respondents (*N* = 51 out of 60) and everyone who is self-employed (see Table 7) earned the minimum study income (US$1.36), compared with men (*N* = 33 out of 50). Men with disabilities earned significantly higher income than women with disabilities. The Mann-Whitney U test, which compared the difference in their income was significant, *Z* = –2.66, *p* = .008. This is expected, given that more than half of the women who were employed worked for themselves. Only a few of the individuals working in the government (5; 8.1%) and private (5; 8.1%) sectors earned the minimum income. The minimum study income, however, is relatively distributed among respondents in all three study locations; with just a few more persons with disabilities (30; 36.2%) from Tamale earning that income compared to the other two study locations, Bolga (27; 32.5%) and Wa (26; 31.3%). There is a similar trend of income distribution among the disability types, with slightly more individuals with physical disabilities (30; 35.7%) earning this income compared to those with hearing (29; 34.6%) and visual disabilities (25; 29.7%). The Kruskal-Wallis test, which compared the income in the three study locations and the disability types, shows that the difference was not statistically significant.

Additionally, there was a significant difference in the income that participants working in the government and private sectors and the self-employed earned. The Kruskal-Wallis test was significant, *x*^2^ (2, *N* = 81) = 48.75, *p* = .000. A three-way Mann-Whitney U was conducted as follow-up test between the three groups. The results of all three the tests, between government and private sectors (*Z* = –2.191, *p* = .036), government sector and self-employed (*Z* = –7.011, *p* = .000), and private sector and self-employed (*Z* = –3.64, *p* = .000) were significant. Participants working in the government sector earned more income than those in the private sector and the self-employed. Also, those working in the private sector earned more income than those who were self-employed.

A few respondents (7; 6.7%) were in the highest monthly income bracket of the study (GhC 401–1100). And virtually everyone earning this income is a male with a disability (6; 85.7%) who worked in the government sector, except one. This person worked in the private sector and, she was the only female of the study who earned that income. It is noteworthy that none of those who earned the highest study income was self-employed.

Asked about their current sources of income, respondents indicated by working for the government, private sector, self-employment and from family and friends as well as begging. Six declined to answer this question. The majority (13; 86.7%) of those who indicated their source of income was their family or friends were unemployed, but (2; 13.3%) were employed. Among those who indicated they begged for a living, the majority (8; 88.9%) were individuals with visual disabilities, one was an individual with a physical disability, but none had hearing disabilities. Also, over three-quarters (7; 77.8%) of those who begged indicated they were unemployed, but about a quarter (2; 22.2%) said they were employed.

### Support

Respondents reported having received financial and non-financial (e.g. food, housing, clothing, and emotional) support from various sources, including the family, friends, non-governmental organizations, churches, mosques and the government both in the past and the present. See Tables 9–10 for more details.

**TABLE 9:** Sources of past support by sex (%) (*N* = 97 Females 53 and Males 44)**.**

Source of past support	Sex			
	Male (%)	Female (%)	Total (%)	*N*
Government	25	24.5	24.7	24 (M=11, F=13)
Family	52.3	50.9	51.5	50 (M=23, F=27)
Friends	11.4	17	14.4	14 (M=5, F=9)
Churches	6.8	3.8	5.2	5 (M=3, F=2)
Mosques	2.3	0	1	1 (M=1, F=0)
NGOs	2.2	3.8	3.3	3 (M=1, F=2)
Total	100	100	100	-

**TABLE 10:** Sources of present support by sex (*N* = 73 Females 41 and Males 33).

Source of present support	Sex			
	Male (%)	Female (%)	Total (%)	*N*
Government	3	2.4	2.7	2 (M=11, F=13)
Family	42.4	53.7	48.6	36 (M=14, F=22)
Friends	27.3	36.3	32.4	24 (M=9, F=15)
Churches	21.2	7.3	13.5	10 (M=7, F=3)
Mosques	3	0	1.4	1 (M=1, F=0)
NGOs	3	0	1.4	1 (M=1, F=0)
**Total**	**100**	**100**	**100**	**-**

However, many respondents (98; 90.6%) said they received more support in the past compared to the present (73; 74.5%). In both periods, respondents indicated that their families were their main source of support. They noted that they received from their families basic needs such as food, shelter, clothing and personal assistance to complete errands. The percentage of participants receiving this support dropped [past 51.5%; present 48.6%].

Government support was the second highest form of support in the past, but that also drastically dropped from 24.7% to 2.7%. An interesting pattern is that support from friends, mostly, non-monetary (personal assistance to complete errands and moral support) more than doubled the past year’s (past [14.4%]; present [32.4%]). Current support from churches (1.4%) and NGOs (1.4%) was very minimal. Another fascinating result is that more women with disabilities than men received support in both eras but this was not statistically significant.

Asked to rate the quality of the current support they received, respondents highly rated government support as very good or good (64.7%) than other sources of support, NGOs (60%), family (57.7%), churches (50%) and friends (47.1). Respondents support needs were identified as: (1) government (for skills training, start-up capital for small businesses, and accommodation on the job, further education, education for their children, and marketing products); (2) family and friends (social support, personal assistance, financial/in-kind support for basic needs); (3) churches (social support, support for children’s education, financial/in-kind support for basic needs); and (4) NGOs (start-up capital/skills training, support for children’s education, financial/in-kind support for basic needs).

Respondents were asked to indicate which of their support need is most important. Over half (65.5%) mentioned government support compared with NGOs (12.4%), family (9.5%), friends (3.8%), and (2.9%) each for churches and mosques. Participants believed that government support as indicated above will give them the independence they so much desire.

## Discussion

This exploratory study establishes relationship among disability, gender and employment. Compared to their male counterparts, the results indicate that women with disabilities have higher unemployment rates, validating studies that women with disabilities are less likely to be employed (Dhungana 2006; Randolph & Anderson 2004; Smith 2007). However, the study outcomes also demonstrate that women with disabilities have shorter lengths of unemployment than men. This is because, as discussed elsewhere in this paper, women are more likely than men to engage in vulnerable employment, working in marginal and seasonal jobs (e.g. selling few groceries on a table, making and selling cooked food, selling smaller bags of produce – usually at home or in front of the house).

Although not statistically significant, women with disabilities experience more of the barriers to the employment of persons with disabilities identified in this study than their male counterparts: (1) discrimination, (2) lack of start-up capital and (3) inadequate skills. It is important to note that discrimination was the key barrier to the employment of persons with disabilities in this study. Discrimination is basically due to preconceptions about their capabilities, supporting other studies (Heymann *et al.* 2013; Mizunoya & Mitra 2012; World Health Organization Report on Disability 2011).

Interestingly, contrary to the SINTEF and other studies (Mitra *et al.* 2011; Mizunoya & Mitra 2012; World Health Organization Report on Disability 2011), neither inadequate skills nor formal education significantly impact the employment of persons with disabilities in this study. The majority of study participants (including those who have no formal education) already had vocational or technical training and could be self-employed. But, they need money to rent places, buy equipment and materials to start and grow their businesses. Hence, start-up capital (an important element to self-employment) remains another important obstacle for the employment of persons with disabilities. This finding is consistent with the World Health Organization Report on Disability (2011:247–248) and the study by Heymann *et al*. (2014). The World Health Organization Report on Disability also indicates that women with disabilities are particularly disadvantaged to start-up capital due to the lack of collateral security, which is a requirement of many banks for loans.

Accordingly, study participants recommended start-up capital as crucial for self-employment. For employment in all sectors (public, private and self-employment), public education about the capabilities of persons with disabilities and the need to end discriminatory practices against them are essential. Although formal education did not appear to be a barrier to the employment of persons with disabilities in this study, they nevertheless recommended improvement in their educational status for employment in both the private and public sectors, which is consistent with the World Health Organization Report on Disability (2011). It is worthy to mention that over a third of the respondents had either no formal education (16; 24.5%) or elementary/junior high education (20; 18.2%) and only a few (8; 7.3%) had undergraduate education. However, it is notable that the educational levels of males with disabilities were significantly higher than their female counterparts, confirming reports by the Food and Agricultural Organisation (FAO 2012) regarding literacy rates; women (46%) and men (67%) in the general Ghanaian population.

Findings also indicate that more than half of persons with disabilities who are employed work for themselves, a phenomenon the United Nations describes as vulnerable employment (United Nations 2013a). Vulnerable employment is characterised by low income, lack of job security and lack of job-related benefits. This finding is consistent with Groce et al.’ s (2013) result about the employment of persons with disabilities in nine developing countries. Women with disabilities dominate the self-employment sector compared to their male counterparts. The vulnerable employment gender gap for this study is rather large (18.6%) compared to the world average (2.3%) and estimates for the Sub-Saharan Africa (15%), (United Nations 2013a), but smaller than the Ghana national average (33.4%) (Ghana Statistical Service 2006). This finding is consistent with Mizunoya & Mitra’s results (2012) which indicate that 9 out of the 15 countries investigated showed significant employment differences between persons with disabilities who are self-employment and their counterparts with no disabilities.

Women with disabilities in this study mostly worked in traditional women’s jobs such as dressmaking, weaving, hairdressing, and petty trading (e.g. selling few groceries on a table usually at home or in front of the house, selling cooked food, smaller bags of produce), thus participating in both the production and service sectors. But, compared to their male counterparts, the women are overrepresented in the production sector; making doormats, baskets, kente clothes and clothing. The results, on the other hand, suggest that men with disabilities dominate the public and private sectors, working mostly in the service industry as teachers. Thus, this study suggests that in Ghana, men with disabilities, rather than women, are overrepresented in the service sector contrary to the literature, but both men and women with disabilities are underrepresented in official and managerial positions.

The study further demonstrates that in Ghana there is a trend in the kinds of jobs individuals with various types of disability do. Those with physical disabilities are more likely to work for themselves, while people with visual disabilities teach in the schools for people with visual disabilities and as craft instructors in regular schools. Individuals with hearing disabilities are more likely to teach in the schools for people with hearing disabilities, but they also work in the private sector, mostly in jobs that require more physical efforts than verbal communication. This is due to the lack of accommodation of specifically sign language interpretation. A further key finding is that the government is the major employer of persons with disabilities, other than those who are self-employed, but they all work in the education sector as teachers.

The study also reveals that persons with disabilities who worked received training on the job to upgrade their skills. However, twice as many men received training compared to women, validating studies that gender and disability interact to create unequal opportunities for men and women with disabilities (Emmett & Alant 2006; Smith 2007). Not many get promoted, confirming studies that persons with disabilities who are employed mostly have difficulties climbing the organizational ladder to work in higher level positions (Barisin *et al.* 2011; Kaye 2009), about twice as many men with disabilities compared to women get promoted.

Persons with disabilities who work experience problems at work, irrespective of their sex, disability type and employment sector. These challenges include negative perceptions about their capabilities, discrimination and exclusion. For instance, respondents from the private and government sectors reported experiencing pay discrimination and verbal abuse. Others said they felt they were not involved in the decision-making process. Yet, others indicated lack of accommodation on the job, also consistent with the World Health Organization Report on Disability (2011). For example, the people with visual disabilities said they do not get brailed textbooks and required software to effectively do their work. They sometimes purchase the essential materials or rely on friends and family to read or dictate the textbooks to them. Another instance of desired accommodation regards changes in working hours for those who need it, especially the women.

Employers are usually concerned about the cost of providing accommodation for persons with disabilities. However, The Job Accommodation Network of the Office of Disability Services of the United States of America’s Department of Labor found that, contrary to employers’ fear of high cost of providing accommodation, benefits (such as ‘retaining valuable employees, improving productivity and morale, reducing workers’ compensation and training costs, and improving company diversity’) far outweighs cost (Job Accommodation Network 2014:3). In some cases, accommodation cost almost nothing.

On the whole, the self-employed were happier and more satisfied with their jobs than those who worked in the public and private sectors. This is because they make their own decisions, especially regarding when to go to work and the number of hours to work, but not necessary the income they earned, because they earned significantly less than those who worked in both the government and private sectors. This finding supports the results from Pagan’s study (2009) which examined the use of self**-**employment among people with disabilities in 13 European countries. She found that persons with disabilities are not only more likely to be self**-**employment, but also, self-employment provides flexible working hours, higher levels of job satisfaction than those persons with disabilities who are wage and salary earners.

Although the majority of the participants indicated they were working, study outcome suggests that most of them lived in poverty, given that the average income per day is less than US$1 (GhC 1.47) and the majority (84; 80%) earn income demonstrated to be just on the poverty threshold set by the World Bank. The daily poverty threshold is estimated at US$1.25 and the minimum daily income reported in this study is equivalent to US$1.36. This finding is consistent with the literature indicating that persons with disabilities are more likely to be poor, especially in developing countries (Appiagyei 2006; Kassah 2008; Mitra *et al.* 2011; Naami & Liese 2012; World Health Organization 2011). A study by Mitra *et al.* (2011), shows significant association of disability and multidimensional poverty in about 14 of the 15 developing countries investigated.

It is important to note that study participants had an average of two children (SD = 1.748). The results show that many women with disabilities (42; 58.3%) have given birth to many children compared with men (30; 41.7%) and since the majority of the study participants could not provide for their basic needs, they depended on their families. This finding is expected because of Ghanaian’s belief in the supporting role of the extended family system. Family members, irrespective of disability status, depend on the family system in times of crises. However, studies show that poverty and negative perceptions about disability impact on the familial support persons with disabilities receive (Naami & Liese 2011). Also, the study by Mitra *et al.* (2011) suggests that in Ghana, households with disabilities generally experience lower levels of economic well-being. It is therefore not surprising that respondents rated other sources of support, especially government, higher than familial support. The majority (68.6%) also cited government support, specifically government support in the form of assistance for skills training, start-up capital for small businesses, accommodation on the job (examples are assistive technology), further education and marketing their products, rather than family support, as their most important support need, because they believed that this form of support is more reliable and could help them gain the independence they have been looking for.

In the absence of jobs and social protection, which according to the World Disability on Report is just 1%–2% of the Gross Domestic Product of developing countries, as well as inadequate social support (which is crucial for survival), many unemployed individuals with disabilities beg for survival. This finding is consistent with studies suggesting that persons with disabilities in Ghana are compelled to beg on the streets due to their exclusion from the labour market (Appiagyei 2006; Kassah 2008). From this study, almost all those who beg for a living are individuals with visual disabilities. A few were individuals with physical disabilities, but none had hearing disability. Surprisingly, none of the beggars saw begging as a job contrary to other studies (Groce *et al.* 2013).

Women with disabilities are overrepresented among those earning the minimum daily income reported in this study (US$1.36), which is demonstrated to be on the poverty threshold, validating studies indicating that women with disabilities are more likely to be poor compared to men with disabilities (Dhungana 2006; Mitra 2006). Also, males with disabilities earn significantly higher incomes than their female counterparts, validating the World Health Organization Report on Disability (2011) and Ozawa & Yeo (2006). Poverty among persons with disabilities is relatively distributed in the three study locations. The proportion of poor persons with disabilities in the Northern Region is slightly higher, (although not statistically significant) compared to the other two regions (Upper East and West). This finding is also not surprising, as the Northern Region is classified as the poorest region in the country (Ghana Statistical Service 2008).

## Recommendations and conclusion

This study, like every other study, has limitations. The results cannot be generalised to the entire population of persons with disabilities in the Northern Sector of Ghana, and in Ghana as a whole, due to use of non-probability sampling method. The sampling method used, recruiting participants from the disability movements and specific towns might have introduced selection bias to the study. The sample may not represent the population of persons with disabilities in the Northern sector. Additionally, poverty estimates in this study might have been underestimated due to participants’ affiliation to the disabilities movement. This connection might have probably positioned them to be better-off than the typical person with a disability in Northern Ghana. These limitations notwithstanding, the following recommendations could advance the employment of persons with disabilities.

As study participants suggested, public education about their capabilities and the need to end discriminatory practices are crucial for their employment, job security and tenure. Persons with disabilities in Ghana continue to experience underemployment and unemployment. Discrimination is cited as the major impediment to the employment of persons with disabilities as well as a challenge for those who work. This recommendation also supports provision in Article 18 of the UN Convention on the Rights of Persons with Disabilities (CRPD) 2006). Educational interventions such as workshops and discussions to demystify the public’s perceptions about disability, documenting and showcasing success stories of persons with disabilities could be helpful in reducing and/or eliminating negative perceptions about the capabilities of persons with disabilities and discrimination against them. More emphasis should be given to women and girls with disabilities, as they are more marginalized due to sexism and disabilism. These interventions should target individuals and organizations that could use their platforms to continue to raise awareness about disability issues after the trainings and discussions are over. Examples are faith-based organizations, teachers, traditional leaders, employers, the media, the traditional institution, and civil society organizations.

The Government of Ghana should find better ways to disburse and monitor the implementation of the District Assembly Common Fund (DACF) as recommended by Social Enterprise Development Ghana (SEND-GH), a civil society organization in Ghana. In 2005, the government gave a directive instructing all District Assemblies (local governments) to allocate up to 5% of their shares of the common fund for persons with disabilities (Republic of Ghana 2009). However, in its quest to support the development of persons with disabilities, the government in 2007 added a ‘ring fencing’ clause to the guidelines for the utilization of the DACF. Part I, guideline #6 of the DACF states that, ‘[t]wo percent (2%) shall be utilized to support initiatives by the physically challenged in the District’ (Republic of Ghana 2009). Nevertheless, a research by SEND-GH (Andoh 2014) indicates that the District Assemblies sometimes borrow from the DACF and do not repay into the fund. This impacts on access of persons with disabilities to the fund. The DACF could be a better source of start-up capital for persons with disabilities, given that the lack of it was identified as an impediment to self-employment. It could also be a source of funding for the self-employed to grow and maintain their businesses, since they are less likely to access other sources of funding due to the lack of collateral security (which is a requirement of financial institutions) as well as discrimination. Additionally, specific guidelines delineating detailed activities (e.g., starting, maintain, growing small businesses, and further education) eligible for funding under the DACF is recommended.

The government could give directives, as in the case of the DACF, to the Microfinance and Small Loans Center to allocate a certain percentage of its funding to persons with disabilities. This regulation may minimize funding inequity for persons with disabilities. The Microfinance and Small Loans Center is a government organization dedicated to giving microfinance services targeted at reducing poverty and creating jobs and wealth. Microfinance is an important tool for poverty reduction and given that many study participants live in poverty, specifically targeting them in the allocation of microfinance credit could reduce their poverty. More attention should be paid to women and girls with disabilities since they suffer poverty the most.

The government must also implement measures to promote equality in the recruitment, training, tenure, promotion and other working conditions of persons with disabilities. It is also important to ensure inclusive and accessible work environment including reasonable accommodation. These are provisions clearly outlined in the CRPD Article 27 well as Article 5 of the CRPD which obliges states to promote equality and eliminate discrimination based on disability. However, just like the case of Tunisia’s disability legislation as cited in Lord *et al.* (2012), Ghana’s disability legislation is also unclear about what reasonable accommodation is. Hence the need to amend the policy document to include this provision.

Although formal education did not appear to be a barrier to the employment of persons with disabilities in this study, participants suggested the need for the government to create educational opportunities for them. This recommendation is consistent with the World Report on Disability (2011). This suggestion is also important given that over a third of the respondents had either no formal education 16 (24.5%) or just elementary/junior high education 20 (18.2%) and only a few 8 (7.3%) had undergraduate education. Currently, the *Persons with Disability Act 715* (Government of Ghana 2006b) makes provisions for free education of persons with disabilities but it is silent about the levels of education covered under this law (basic?, high school? or college levels?). It is imperative that the state adhere to the provisions made in Article 24 of the CRPD which requires states to provide free access to education for persons with disabilities at all levels without discrimination on the basis of disability. Ghana is bound by all provisions in this document as the state ratified this convention in July 2012. The government must also provide reasonable accommodations at all levels as required by each individual and the failure to do so will amount to discrimination which contravenes this provision. This also means training and employing teachers who are qualified to effectively include individuals with disabilities in the education system. For examples, teachers who can ‘facilitate the learning of braille, alternative script, augmentation and alternative modes, means and format of communication and orientation and mobility skills …’ as stated in Article 24(3a) of the CRPD. More attention should be given to girls and women with disability to ensure they also enjoy these provisions because they are more likely to experience multiple discrimination, as noted in Article 6 of the CRPD.

The educational environment must be free of physical barriers which could impact the educational outcome of persons with disabilities. The need to fix ramps and elevators to buildings as well as sidewalks, curb cuts and zebra crossings cannot be overemphasized. Also, as accessible transportation is more likely an important element for positive educational outcome, the government must make conscious efforts to ensure that the transportation system in Ghana is accessible for persons with disabilities. These recommendations build on Article 9a of the CRPD which requires buildings, roads, transportation, housing, schools and other facilities made accessible for persons with disabilities.

The government should develop welfare policies and programs for the children of persons with disabilities. This is necessary, as over half of the participants have children they cannot provide for due to their poverty situation. Such programs are more likely to advance on the health, educational outcomes and overall well-being of the children of persons with disabilities and of persons with disabilities as a whole.

There is also a need for the government to develop measures to collect data about persons with disabilities as indicated in Article 31 of the CRPD. Lack of information about disability, which is the current situation, impact appropriate policies and programs that could benefit persons with disabilities. Disagregated data is recommended to effectively address issues specific to the disability groups.

For future research, there is a need to collect data that is nationally representative of persons with and those without disabilities and research to comparing these two populations to better understand the relationships among disability, gender and employment.
